# Time and Energy Efficient Relay Transmission for Multi-Hop Wireless Sensor Networks

**DOI:** 10.3390/s16070985

**Published:** 2016-06-27

**Authors:** Jin-Woo Kim, José Ramón Ramos Barrado, Dong-Keun Jeon

**Affiliations:** 1 Research Institute of Information Science and Engineering, Mokpo National University, Mokpo city 530-729, Korea; jjin300@gmail.com; 2 Department of Applied Physics I, University of Malaga, Avenida Cervantes, 2, Málaga 29071, Spain; jdk0076@hanmail.net; 3 Department of Mechatronics, Incheon National University, Incheon city 406-772, Korea

**Keywords:** Internet of Things, IEEE 802.15.4, relay communication, wireless sensor network

## Abstract

The IEEE 802.15.4 standard is widely recognized as one of the most successful enabling technologies for short range low rate wireless communications and it is used in IoT applications. It covers all the details related to the MAC and PHY layers of the IoT protocol stack. Due to the nature of IoT, the wireless sensor networks are autonomously self-organized networks without infrastructure support. One of the issues in IoT is the network scalability. To address this issue, it is necessary to support the multi-hop topology. The IEEE 802.15.4 network can support a star, peer-to-peer, or cluster-tree topology. One of the IEEE 802.15.4 topologies suited for the high predictability of performance guarantees and energy efficient behavior is a cluster-tree topology where sensor nodes can switch off their transceivers and go into a sleep state to save energy. However, the IEEE 802.15.4 cluster-tree topology may not be able to provide sufficient bandwidth for the increased traffic load and the additional information may not be delivered successfully. The common drawback of the existing approaches is that they do not address the poor bandwidth utilization problem in IEEE 802.15.4 cluster-tree networks, so it is difficult to increase the network performance. Therefore, to solve this problem in this paper we study a relay transmission protocol based on the standard protocol in the IEEE 802.15.4 MAC. In the proposed scheme, the coordinators can relay data frames to their parent devices or their children devices without contention and can provide bandwidth for the increased traffic load or the number of devices. We also evaluate the performance of the proposed scheme through simulation. The simulation results demonstrate that the proposed scheme can improve the reliability, the end-to-end delay, and the energy consumption.

## 1. Introduction

A wireless sensor network is a kind of new wireless network that transmits sensing data to a destination device. Devices in the wireless sensor network operate on battery power and at low data rate since it is generally impossible to connect them to a power wire.

The IEEE 802.15.4 standard is a new standard protocol for low-power and low-rate WPANs (wireless personal area networks) that defines the physical (PHY) and media access control (MAC) layers [1,2]. The IEEE 802.15.4 standard can configure the wireless network using PHY and MAC layers and can provide a network service such as ZigBee and 6LoWPAN to a higher layer [3,4,5]. The upper layer standard of the IEEE 802.15.4 standard defines a communication scheme between source devices and destination devices and can be applied to various applications. In particular, the IEEE 802.15.4 and ZigBee standards are suitable for wireless sensor networks that require low power and lightweight communication solutions.

The IEEE 802.15.4 standard configures the WPAN by connecting each device around the PAN coordinator, and the WPAN can include various sensor devices or coordinators. When a link quality between the PAN coordinator and an end device is good enough, the PAN coordinator can communicate with the end device directly. However, because sensor nodes are designed to operate on low power, they should communicate with each other using the limited transmission power and have problems such as a limited communication coverage. If the sensor device is out of communication range of the PAN coordinator, an auxiliary device needs to relay data frames between the PAN coordinator and the end device. In general, a routing protocol serves this multi-hop transmission. When the number of hop between the PAN coordinator and the end device increases, the transmission delay increases, and the network performance degrades. Also, because the existing routing protocols require more processing for multi-hop transmission in the network layer, they consume more energy. To address this problem, various approaches have been developed to maintain the energy efficiency at every layer of the protocol stack by offering new algorithms and protocols [6,7,8,9]. However, the separation of layers has become an obstacle to improving the network performance. Also, when the IEEE 802.15.4 standard is extended to a multi-hop wireless network, all nodes in the network have to operate on the same duty cycle, and the number of nodes belonging to the same WPAN is limited.

Also, in the cluster-tree topology of the legacy IEEE 802.15.4 standard, the coordinators must compete against their children devices to relay data frames. Therefore, when the traffic load or the number of devices increases, the network performance also decreases. Also, the IEEE 802.15.4 cluster-tree topology may not be able to provide sufficient bandwidth for the increased traffic load or the number of devices and may not deliver the additional information to manage the network successfully. The common drawback of the existing approaches is that they do not address the poor bandwidth utilization problem in IEEE 802.15.4 cluster-tree networks, so it is hard to increase the network performance. Therefore, to solve this problem, we propose new relay transmission scheme to reduce the transmission delay time and the energy consumption in the IEEE 802.15.4 cluster-tree networks. Also, we propose the enhanced beacon scheduling scheme to extend the coverage of wireless sensor networks and to optimize the wireless resource allocation.

## 2. Background

### 2.1. Overview of IEEE 802.15.4 Standard

**Figure 1:**
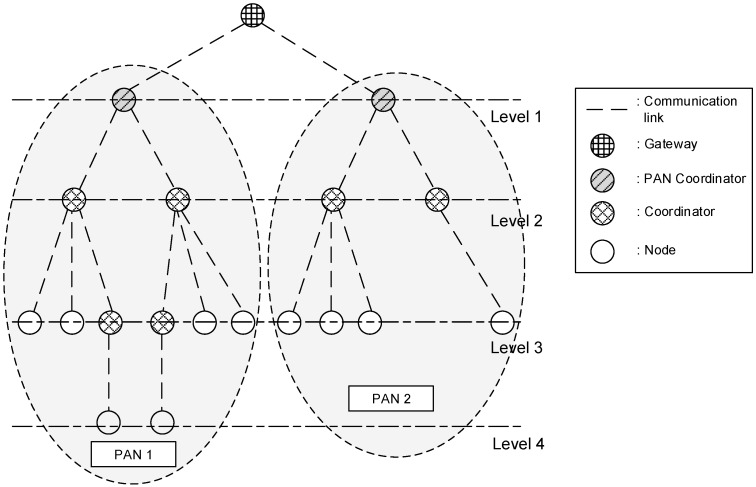
An example of a sensor network with a cluster tree topology.

The IEEE 802.15.4 protocol is the international standard for a wireless sensor network and defines the PHY/MAC layer for the wireless sensor network and the interface with the network layer for the wireless sensor network applications. The IEEE 802.15.4 standard supports the offset quadrature phase-shift keying (O-QPSK) PHY, which employs a 16-ary quasi-orthogonal modulation technique. The data rate of the O-QPSK PHY shall be 250 kbps when operating in the 2.4 GHz band. When the PAN coordinator builds a WPAN, it periodically broadcasts its beacon frame after it selects a channel for its operation. Then, when a sensor device receives the beacon frame, it transmits an association frame to the PAN coordinator to join the WPAN. Figure 1 shows an example of a sensor network with a cluster tree topology.

In Figure 1, all devices in the wireless sensor network can connect to the Internet through a gateway. Also, PANs 1 and 2 can extend to the cluster tree topology. Namely, there can exist multiple PANs in the same wireless channel environment. The PAN coordinator connected to the gateway manages its WPAN and can be connected to multiple devices and coordinators. Also, all coordinators can be connected to multiple devices and other coordinators.

**Figure 2:**
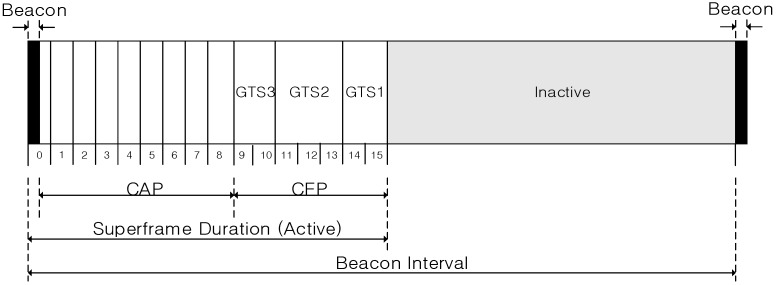
The structure of the superframe of the IEEE 802.15.4 standard.

Figure 2 shows the structure of a superframe defined in the IEEE 802.15.4 standard.

In Figure 2, the superframe of the IEEE 802.15.4 standard is divided into three parts, a beacon period, a superframe duration (SD), and an inactive period. In the beacon period, the PAN coordinator broadcasts its beacon frame, and all devices in the WPAN communicate with each other in the SD. Also, in the inactive period, all devices in WPAN enter into a sleep mode or a standby mode to reduce an energy consumption. The beacon period is located at the beginning of the superframe, and a beacon interval (BI) is equal to the length of one superframe.

The SD of the superframe of IEEE 802.15.4 standard is divided into a contention access period (CAP) and a contention free period (CFP). In the CAP, all devices in the WPAN can communicate with each other anytime. However, if multiple devices transmit frames at the same time, collisions occur in the corresponding time slot. Contrary to the CAP, in the CFP, only allowed devices can transmit data frames in the time slot which is allocated by the PAN coordinator. Thus, collisions among devices do not occur in the CFP.

As mentioned above, the PAN coordinator can communicate with multiple coordinators on the same channel. Each coordinator can broadcast its beacon frame independently and configure its superframe. To put this more concretely, the SD of each coordinator has to locate in the inactive period of the PAN coordinator. Otherwise, there can occur collisions between member nodes of the PAN coordinator and member nodes of the coordinator.

In Figure 1, coordinators can work as relay nodes between the PAN coordinator and sensor devices in the inactive period of the PAN coordinator. However, when the number of coordinators increases, the available resources in the superframe decrease. Furthermore, when the coordinator works as the relay device, the available SD of the PAN coordinator is reduced by half. This feature of IEEE 802.15.4 standard may limit the number of member devices in the WPAN and obstruct the extension of the network coverage. Therefore, in this paper, we propose new relay transmission scheme to expand wireless network coverage and to optimize the wireless resources of the wireless sensor network. To address these issues, we propose a new superframe structure with variable CAP and CFP.

### 2.2. Related Works

In recent years, there has been an increasing interest in wireless networks. Since wireless nodes are usually energy constrained, the energy efficiency of a wireless network is an essential requirement to maximize the lifetime of the entire network. In [10], the authors have classified the major sources of energy waste in wireless networks such as collisions, overhearing and idle listening. In this paper, we propose new relay transmission scheme to eliminate the sources of the energy waste by using time division cluster scheduling and GTS mechanism.

Network scalability is an important requirement in a wireless sensor network. Thus, there have been several research works aimed at improving network scalability. A real-time routing algorithm minimizing energy consumption was proposed in [11], where the authors have assumed a collision-free MAC protocol, and they have used a multicommodity flow model to schedule the optimal flows’ paths in terms of energy consumption while not exceeding links’ bandwidths. The routing algorithm ensures polynomial-time complexity but no scheduling is considered. In this paper, we present a cluster-tree routing protocol where the routing paths are unique, and the routing decisions are simple and time efficient. Our work also utilizes short addresses for routing decisions of relay nodes. The advantage of using short addresses for relay nodes is that they do not need to maintain routing tables to forward incoming data, since by simply looking at the destination address, they can decide to forward the packet upwards (to a parent) or downwards (to a child) [12]. Koubaa et al. [13] proposed an algorithm for collision-free beacon scheduling in IEEE 802.15.4 cluster-tree networks, using the time division approach. In [13], nodes implement a distributed scheduling algorithm so that interfering coordinators maintain non-overlapping superframes. While a pure time-division approach solves the beacon frame collision problem, it limits the network scalability regarding the number of clusters. In [14], the authors proposed an algorithm to adjust the resource allocation for the cluster tree topology on the fly. The authors proposed this algorithm to apply to applications which deliver data to the root of the tree. However, this algorithm is not useful in the case of simultaneous flows in opposite directions.

One of the most significant current topics of discussion regarding wireless networks is cooperative communication. The idea of cooperative communication is to improve the performance and coverage of the wireless network with the support of a relay node. Recently, various studies have been carried out with cooperative communication. Notably, early research on cooperative communication mainly focused on physical layer [6,7,8,9,15,16,17,18,19,20,21]. However, a lot of research is still needed for a practical high layer protocol to realize effective cooperative communications. From a physical layer’s point of view, the source simply broadcasts its data and does not need to know about the relay node. However, from a higher layer’s point of view, a link between the source node and the destination node should be established for non-broadcast services. Also, to select the relay node, the higher layer needs the information for neighbor devices. Different MAC protocol designs supporting cooperative communication among nodes are discussed and compared in [22,23], where the corresponding authors proposed cooperative communication schemes to mitigate the throughput bottleneck caused by low-data-rate nodes. To address the throughput bottleneck, in [22,23], a high-data-rate node is enabled to help a low-data-rate node through a two-hop transmission. However, two protocols based on the IEEE 802.11 DCF are not suitable for the IEEE 802.15.4 standard which is managed by the PAN coordinator. Also, two cooperative MAC protocols usually focus on a single-hop network, but the MAC design for the wireless sensor network needs to extend to address a multi-hop link. In [24], authors proposed a cooperative MAC protocol based on TDMA. However, the TDMA-based MAC protocol is not suitable for the IEEE 802.15.4 standard. Also, an energy efficient communication technique for the multi-hop link has been proposed to increase the lifetime of wireless sensor networks [25]. In [25], the authors try to extend the lifetime through adaptive sleep. But the communication scheme proposed in [25] cannot solve high latency by the competition for a channel access and does not consider applying to the IEEE 802.15.4 standard.

There are also numerous interest research on cooperative MAC based on the IEEE 802.15.4 standard [9,26,27,28,29]. However, cooperative schemes in [9,26,27,28] are mainly focused on the PHY layer. Also, the cooperative scheme proposed in [29] is not suitable for the multi-hop network environment since it is proposed for the single hop WPAN environment. Therefore, in this paper, we propose a new cooperative scheme suitable for multi-hop networks.

## 3. Proposed Scheme

**Figure 3:**
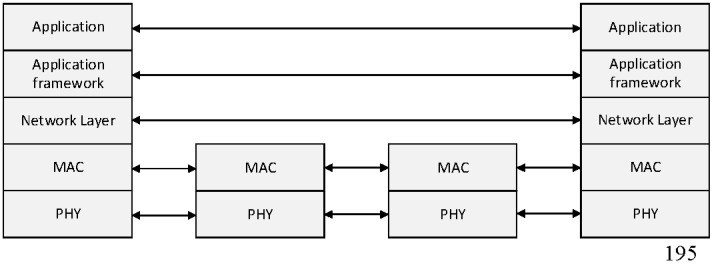
The protocol stack of the proposed relay transmission scheme.

As shown in Figure 1, sensor nodes transmit the sensing data to the coordinator or the PAN coordinator after they obtain sensing data. In the proposed scheme, the coordinator relays data frames received from sensor nodes or low-level coordinators. The PAN coordinator configures its WPAN, manages its member nodes, and processes the received data. Figure 3 shows the protocol stack of the proposed relay transmission scheme.

As shown in Figure 3, MAC and physical layers of all devices are based on the IEEE 802.15.4 standard, and their upper layers are based on the ZigBee standard. In the proposed scheme, the coordinator uses only the IEEE 802.15.4 standard to forward sensing data.

In the current IEEE 802.15.4 cluster-tree topology, the PAN coordinator and intermediate coordinators must compete against their children devices to deliver data frames to the previous or next coordinator. Therefore, when the traffic load or the number of devices increases, the coordinator may take several BIs to deliver data frames to the next coordinator. If these phenomena continue for a long time, the network performance significantly decreases. Also, the IEEE 802.15.4 cluster-tree topology may not be able to provide sufficient bandwidth for the increased traffic load or the number of devices and may not deliver the additional information to manage the network successfully. The common drawback of the existing approaches is that they do not address the poor bandwidth utilization problem in IEEE 802.15.4 cluster-tree networks, so it is hard to improve the network performance. To address these problems, in this paper, we propose a new relay transmission scheme suitable for the IEEE 802.15.4 cluster-tree topology. The proposed scheme provides the RP to deliver data or control frames to the next coordinator without the contention for the channel access. Also, the proposed scheme includes various time offsets for the distributed beacon scheduling in each beacon frame. Thus, all devices in the network can easily synchronize with their parent coordinators and can easily adapt to the adjusted BI.

### 3.1. Initial PAN Construction

**Figure 4:**
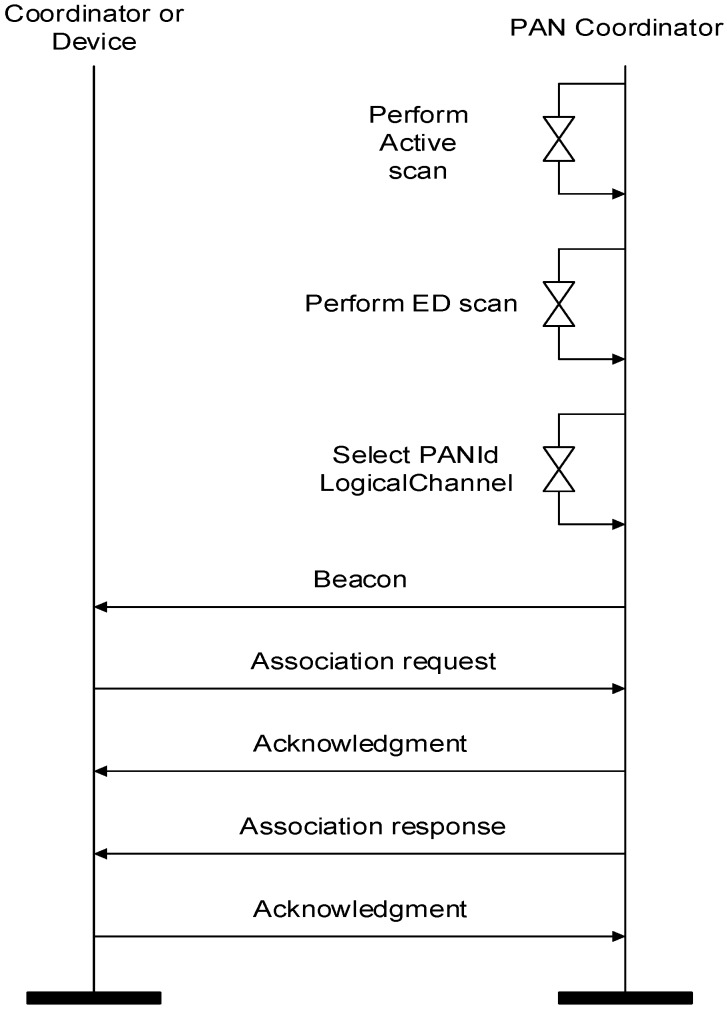
Initial PAN construction flow.

At the initial phase, a PAN coordinator performs an active scan to select a PAN identifier before starting a new PAN, as depicted in Figure 4. Then, the PAN coordinator performs an ED scan to select a clear channel. The PAN coordinator can detect the interference of other networks through the ED scan and save the set of available channels that are not affected by other wireless devices. Then, the PAN coordinator selects a PAN ID and logical channel. The PAN coordinator starts its PAN and commences transmitting periodic beacon frames. A coordinator or devices that receive the beacon frame from the PAN coordinator transmit an association request frame. When the PAN coordinator receives the association request frame, it transmits the association response frame to coordinators or devices. When the devices or the coordinators receive the association response frame from the PAN coordinator, the initial PAN construction process is complete.

**Figure 5:**
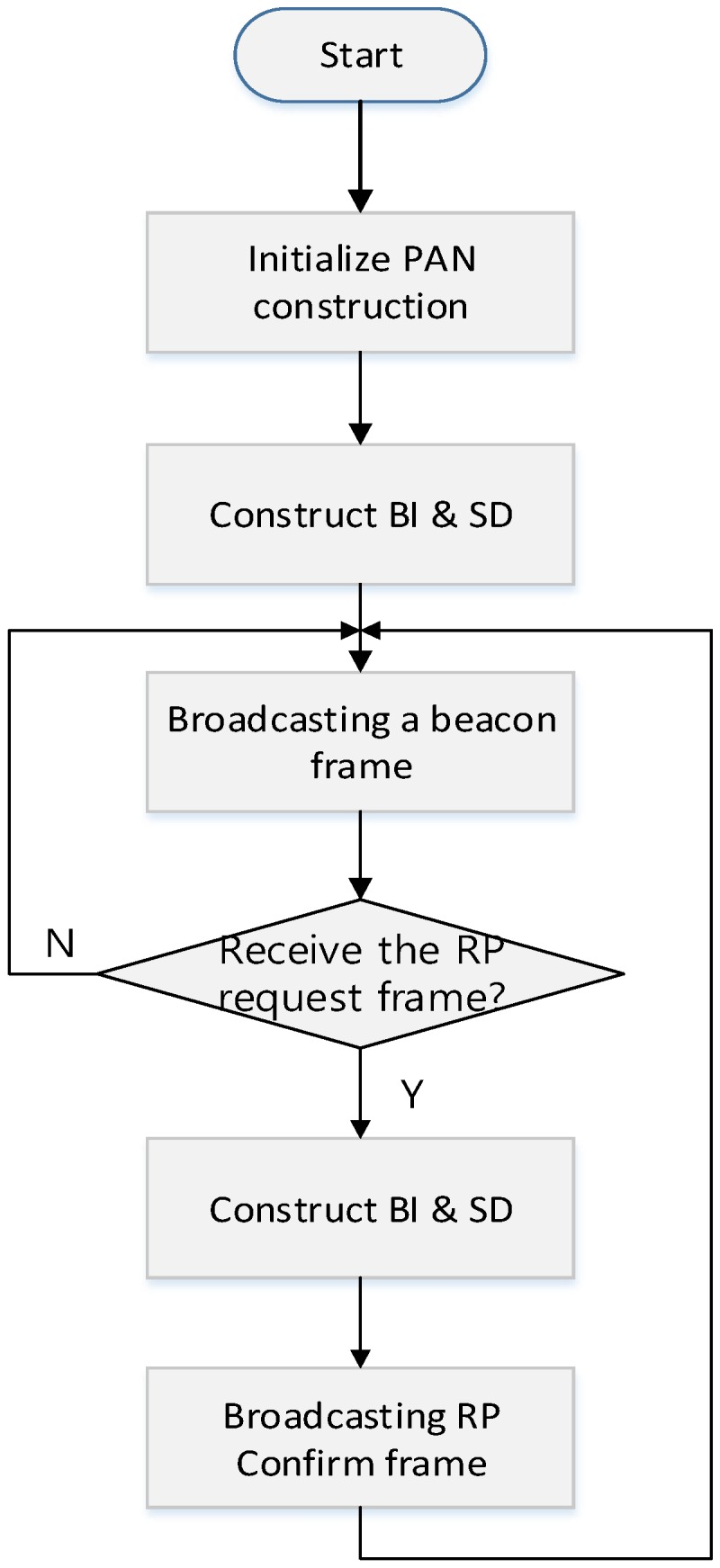
The superframe configuration process for the proposed relay scheme.

Figure 5 shows the superframe configuration process for the proposed relay scheme after the PAN coordinator constructs its own WPAN. In Figure 5, when the PAN coordinator configures its own WPAN, it decides the BI and SD of the superframe and broadcasts the beacon frame including the information of the BI and SD. Devices which receive the beacon frame communicate with the PAN coordinator in the SD. Also, the PAN coordinator monitors whether devices transmit a proposed RP (relay period) request frame or not. When it receives the RP request frame, it allocates a resource for the relay communication in the CFP and broadcasts a proposed RP response frame. If necessary, in the proposed scheme, the PAN coordinator can adjust the length of CAP and CFP.

**Figure 6:**
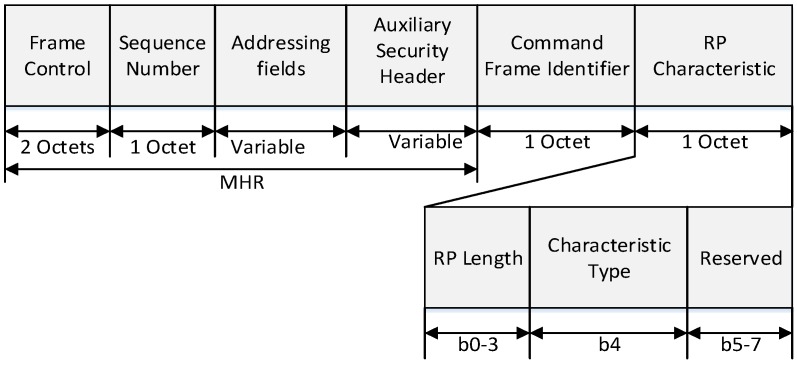
The structure of the proposed RP request command frame.

**Figure 7:**
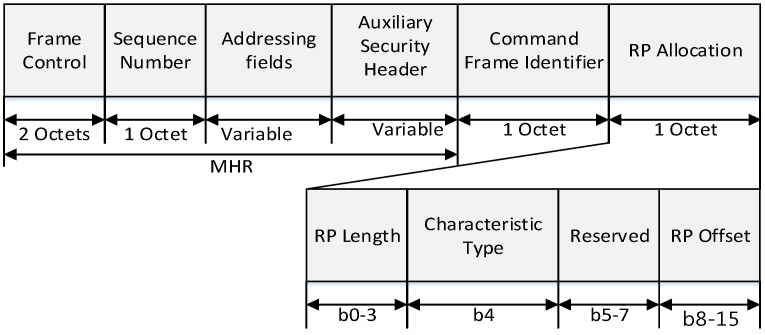
The format of the proposed RP response command frame.

Figure 6 and Figure 7 show the format of the proposed RP request frame and RP response frame.

In Figure 6, the MAC header (MHR) field contains the frame control field, the sequence number field, the addressing field, and the auxiliary security header field. The destination addressing mode field in frame control field is set to indicate the short addressing of destination device, and the source addressing mode field in frame control field is set to indicate the short addressing of source device. The frame pending field in frame control field is set to zero and ignored upon reception, and the acknowledgment request (AR) field in the frame control field is set to one. The source PAN identifier field in the addressing field contains the value of macPANId, and the source address field in addressing field contains the value of the short address of source device. The destination address field in addressing field contains the values of the short address of destination device.

The RP length field contains the number of superframe slots being requested for the RP. The characteristics type field is set to one if the characteristics refer to the RP allocation or zero if the characteristics refer to the RP deallocation.

**Figure 8:**
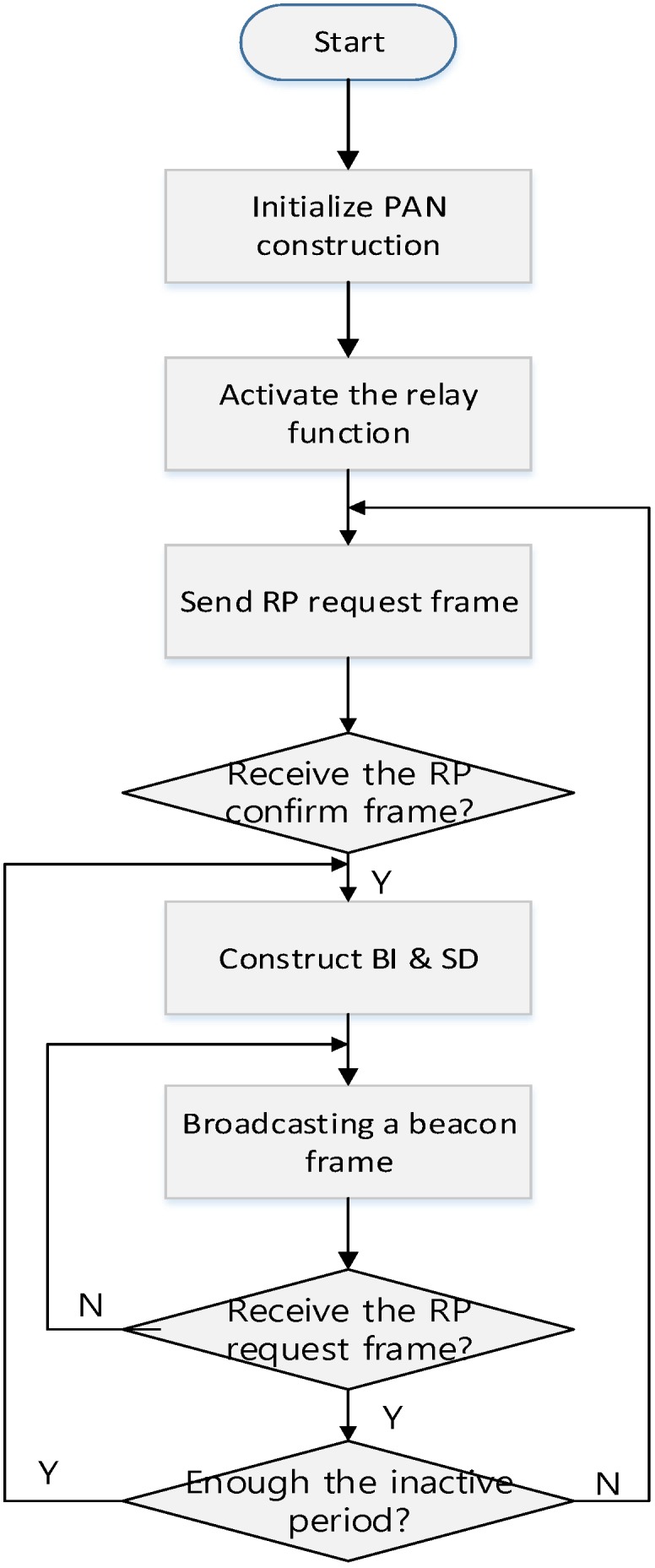
The superframe configuration process by a coordinator.

In Figure 7, the RP length field contains the number of time slots being requested for the RP. The characteristics type field is set to one if the characteristics refer to the RP allocation or zero if the characteristics refer to the RP deallocation. The RP offset field indicates the time slot at the beginning of RP in the SD of the PAN coordinator. Figure 8 shows the superframe configuration process by a coordinator using the proposed relay scheme.

**Figure 9:**
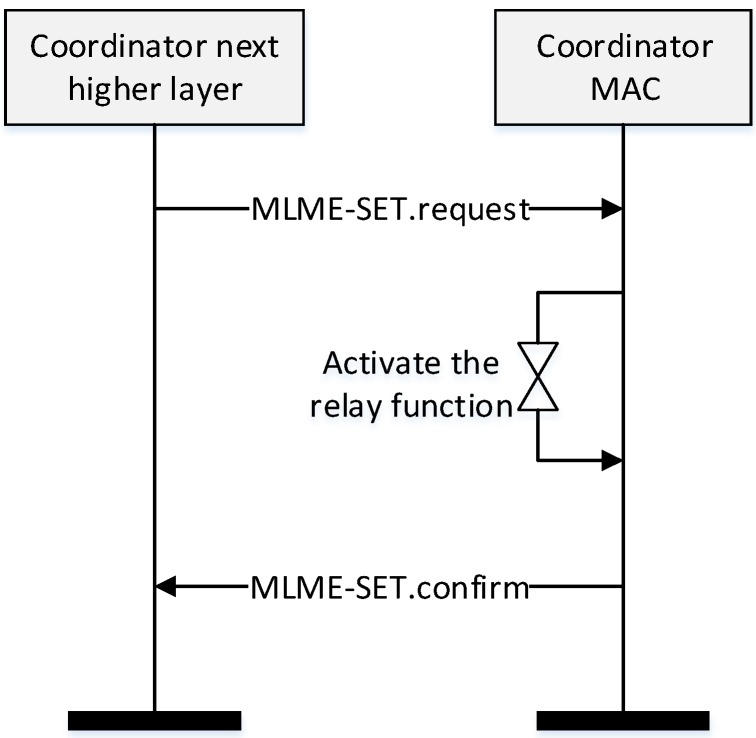
The relay function activation process of the coordinator.

As shown in Figure 8, when a coordinator powers on, it performs the ED scan and joins the existing WPAN. And then, it activates the relay function and transmits the RP request command frame to the PAN coordinator or the higher level coordinator. When it receives the RP response command frame, it constructs its superframe. After that, the coordinator broadcasts a beacon frame including the information of the BI and SD and monitors whether other coordinators transmit the RP request command frame. If it receives the RP request frame, it relays the received RP request frame to the PAN coordinator or the higher level coordinator. In the proposed scheme, the BI of every coordinator in the WPAN is equal to the BI of the PAN coordinator. However, the SD of the coordinator can be adjusted by the number of devices and the lower level coordinators. If the inactive period is insufficient to allocate the SD to the lower level coordinator, the PAN coordinator can adjust its own BI. In the proposed scheme, because all coordinators transmit the beacon frame to their children coordinators in the RP, and the RP is allocated in the CFP, all device can receive the beacon frames from their parent coordinators without the contention. Therefore, the beacon frame transmitted by the PAN coordinator can deliver to all devices in the network without any collision. Also, in the proposed scheme, all devices only synchronize with their parent coordinator and are not affected by other coordinators. Thus, all coordinators can perform the distributed beacon scheduling and can communicate with their children devices in the next SD of their parent coordinator. If the PAN coordinator needs the additional resource and adjusts the BI, every coordinator adjusts their own superframes. Otherwise, the coordinator does not allocate the SD to the lower level coordinator. Figure 9 shows the relay function activation process of the coordinator.

In Figure 9, when the coordinator powers on, its higher layer transmits a MAC layer management entity (MLME)-SET.request message to its MAC layer. Table 1 shows parameters of the proposed MLME-SET.request message.

As shown in Table 1, to turn on/off the relay function, we add the RelayFunction parameter in the MLME-SET.request message and define its type to the Boolean type. If the value of the RelayFunction parameter is set to ‘TRUE’, the coordinator activates the relay function. Otherwise, the ZigBee protocol performs a routing protocol as the usual method. When the MAC layer receives the MLME-SET.request message from the higher layer, the coordinator activates the relay function. And then, the MAC layer transmits the MLME-SET.confirm message to the upper layer and constructs the relay table.

### 3.2. New Superframe Structure for the Proposed Relay Scheme

**Figure 10:**
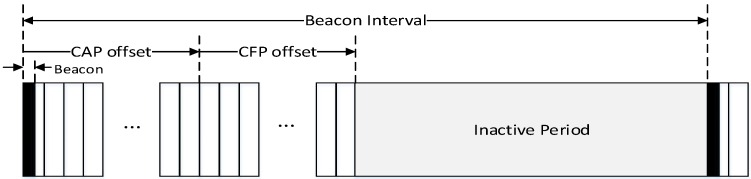
New superframe structure for the proposed relay scheme.

Figure 10 shows novel superframe structure for the proposed relay transmission scheme.

**Figure 11:**
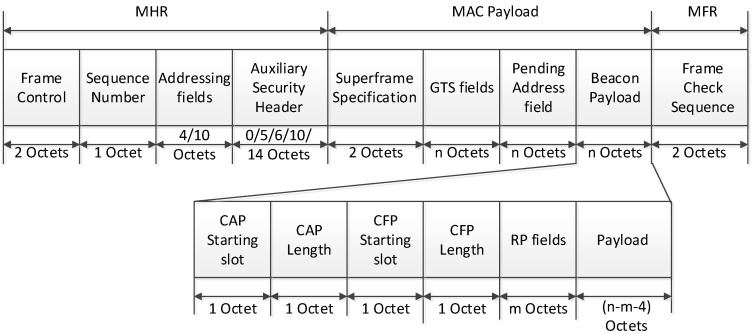
The format of the proposed beacon frame.

In Figure 10, the PAN coordinator periodically broadcasts the beacon frame in the beacon period. The superframe is composed of numerous time slots with the fixed length, and the time slot is the basic unit of a CAP offset and a CFP offset. In the proposed scheme, because the beacon frame includes the information of the CAP offset and the CFP offset, devices which receive the beacon frame can recognize the length of the CAP and the CFP. Figure 11 shows the format of the proposed beacon frame.

**Figure 12:**
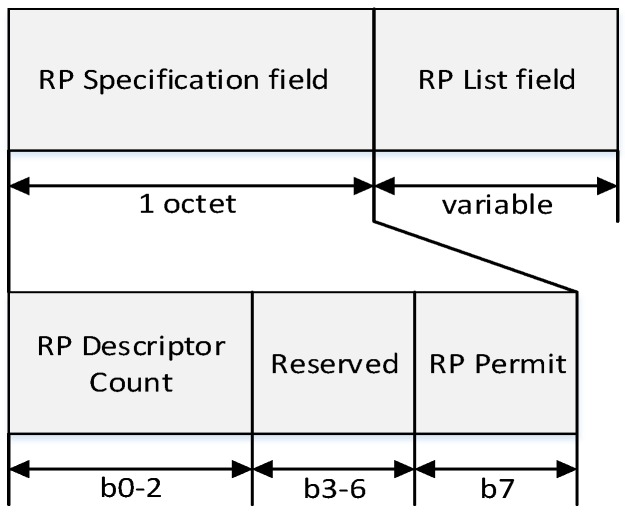
The format of the RP fields.

In Figure 11, the CAP offset is composed of a CAP starting slot field and a CAP length field. The CAP starting slot field indicates the time slot number at the beginning of the CAP, and the CAP length field indicates the number of time slots belonging to the CAP. In the same way, the CFP offset is composed of a CFP starting slot field and a CFP length field. The CFP starting slot field indicates the time slot number at the beginning of the CFP, and the CFP length field indicates the number of time slots belonging to the CFP. The sum of the CAP offset and the CFP offset cannot exceed the value of BI. In the proposed scheme, the PAN coordinator and coordinators can adjust the length of the CAP and the CFP. The RP field contains the information of the RP. Figure 12 illustrates the format of the RP fields.

In Figure 12, the RP descriptor count field specifies the number of RP descriptors contained in the RP List field of the beacon frame. If the value of this field is equal to zero, the RP List field of the beacon frame is not present. The RP Permit field is set to one if the coordinator is accepting RP request. Otherwise, the RP permit field shall be set to zero. The size of the RP List field is defined by the values specified in the RP specification field of the beacon frame and contains the list of RP descriptors that represents the RP that is being maintained.

**Figure 13:**
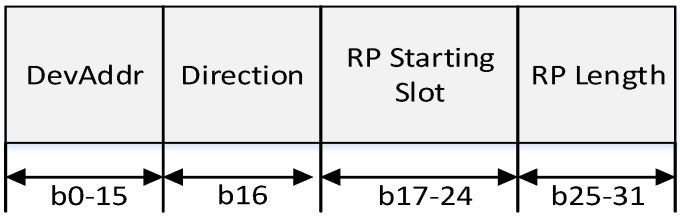
The format of the RP Descriptor.

Figure 13 shows the format of each RP descriptor.

**Figure 14:**
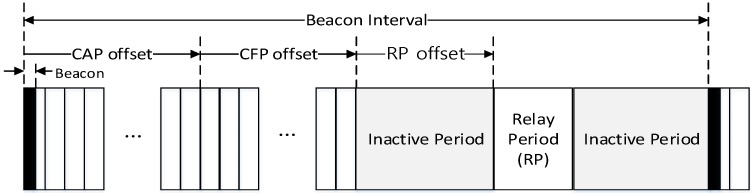
The superframe structure constructed by the coordinator.

In Figure 13, the DevAddr field contains the short address of the device which is allocated the RP from the PAN coordinator or high-level coordinator (parent coordinator). The direction field indicates the direction of the data transmission. If the value of this field is zero, the parent coordinator or the PAN coordinator transmits a data frame to the low-level coordinator (child coordinator) after transmitting the beacon frame. Then, the low-level coordinator transmits the data frame. If the value of this field is one, the low-level coordinator transmits data frame after transmitting the beacon frame. The RP starting slot field indicates the time slot number in which the CFP allocated for the relay communication is begun, and the RP length field indicates the number of time slots belonging to the RP. Figure 14 shows the superframe structure constructed by the coordinator with the relay function.

**Figure 15:**
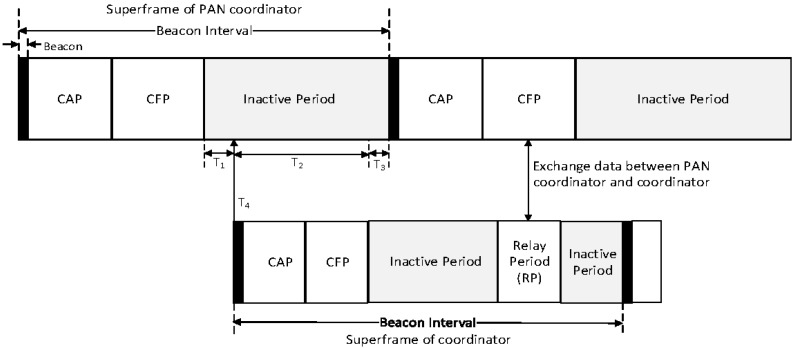
An example of the superframe operation between the high level coordinator and the low level coordinator in the proposed scheme.

In the proposed scheme, the coordinator can connect to the high-level coordinator or the PAN coordinator. Also, it can connect to the low-level coordinator. In Figure 14, the superframe managed by the coordinator consists of the beacon period, the CAP, the CFP, the inactive period, and the RP. The coordinator uses the beacon period, the CAP, and the CFP to communicate with the low-level coordinator and devices belonging to its own WPAN. Whereas it uses the RP to communicate with the high-level coordinator or the PAN coordinator. Namely, the RP is allocated to relay data frames between the high-level coordinator and the low-level coordinator by the high-level coordinator. In the proposed scheme, the sum of the CAP offset, the CFP offset, the RP offset, and the RP lengths cannot exceed the BI length. Figure 15 shows an example of the superframe operation between the high-level coordinator and the low-level coordinator in the proposed scheme.

**Figure 16:**
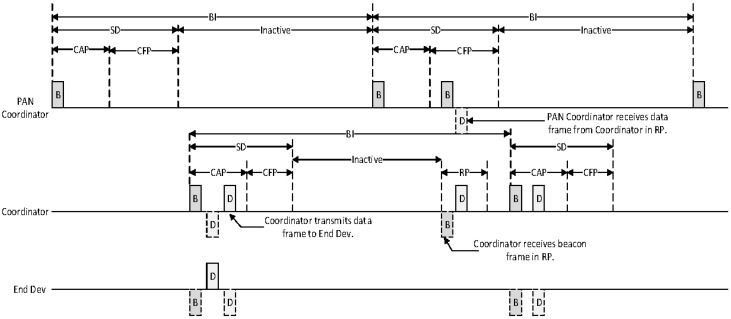
The data flow from the PAN coordinator to the end device of the coordinator.

In Figure 15, the PAN coordinator manages multiple sensor devices and a low-level coordinator, and the low-level coordinator manages multiple sensor devices belonging to its own WPAN. Devices belonging to the PAN coordinator communicate with the PAN coordinator in the SD of the PAN coordinator. As mentioned above, the BI of every coordinator is equal to the BI of the PAN coordinator, and it can be adjusted by the PAN coordinator. As shown in Figure 16, T_4_, which is the beginning of the beacon period of the coordinator, should be the time after T_1_, which is the beginning of the inactive period of the PAN coordinator. Also, to avoid collisions between devices belonging to the PAN coordinator and devices belonging to the coordinator, T_2_, which is the SD of the coordinator, should be smaller than the inactive period of the PAN coordinator (T_3_ > 0).

The coordinator transmits data frames received from devices belonging to its own WPAN to the PAN coordinator in the RP. In this case, the coordinator is allocated the RP to the PAN coordinator and the RP is located in the CFP of the PAN coordinator. Also, to the contrary, the coordinator transmits the received data frame to its members in its SD after receiving the data frame from the PAN coordinator in the RP. Figure 16 shows the data flow from the PAN coordinator to the end device of the coordinator.

As shown in Figure 16, the coordinator communicates with its member in its SD and receives the beacon frame from the PAN coordinator in the RP. Because it receives the beacon frame in the RP, it can synchronize with the PAN coordinator. Also, it transmits the received data frame to the PAN coordinator in the RP after receiving the data frame from its member in its SD.

### 3.3. The Data Transmission in the Proposed Relay Scheme

**Figure 17:**
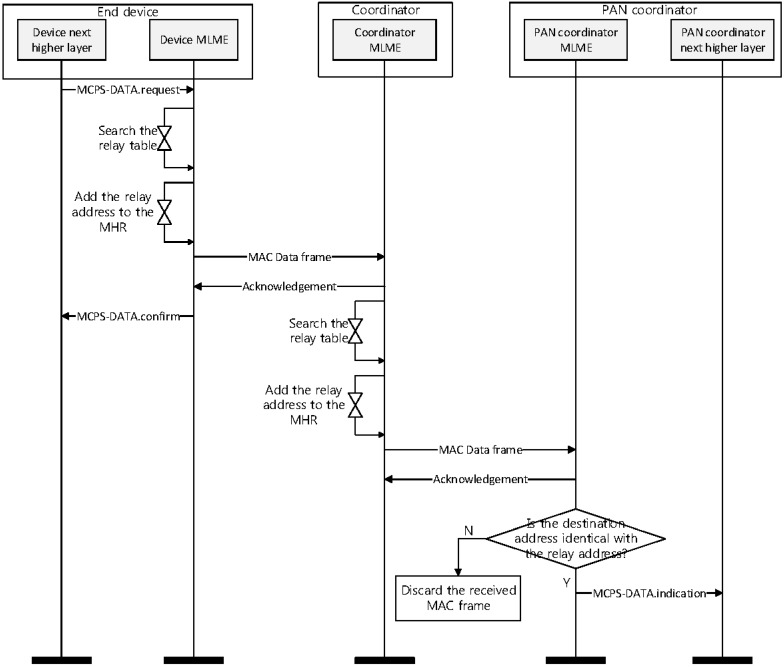
The timing diagram for the proposed relay scheme from an end device to a PAN coordinator.

Figure 17 shows a timing diagram for the proposed relay scheme from an end device to a PAN coordinator.

**Figure 18:**
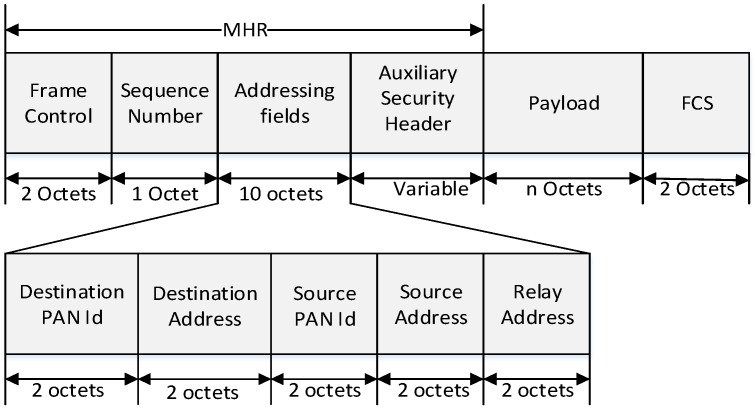
The format of the proposed MAC data frame.

In Figure 17, if an end device gets a data frame, its higher layer sends a MAC Common Part Sublayer (MCPS)-DATA.request message including a sensing data and an address of the PAN coordinator to a MAC layer. When the MAC layer of the end device receives the MCPS-DATA.request message from the higher layer, it searches its relay table. If there exists the address of the PAN coordinator in the relay table, it adds the PAN coordinator address to the addressing fields in a MAC data frame. Figure 18 shows the format of the proposed MAC data frame.

As shown in Figure 18, we add a relay address field to the addressing field for the proposed scheme. The relay address field is utilized to select the coordinator to relay a data frame. The MAC layer of the end device sets the relay address field to the address of the coordinator and the destination address field to the address of the PAN coordinator. Then, it transmits the data frame to the coordinator. When the coordinator receives the data frame from the end device, it transmits an acknowledgement (ACK) frame to the end device. When the MAC layer of the end device receives ACK frame, it transmits an MCPS-DATA.confirm message to the higher layer of the end device.

**Figure 19:**
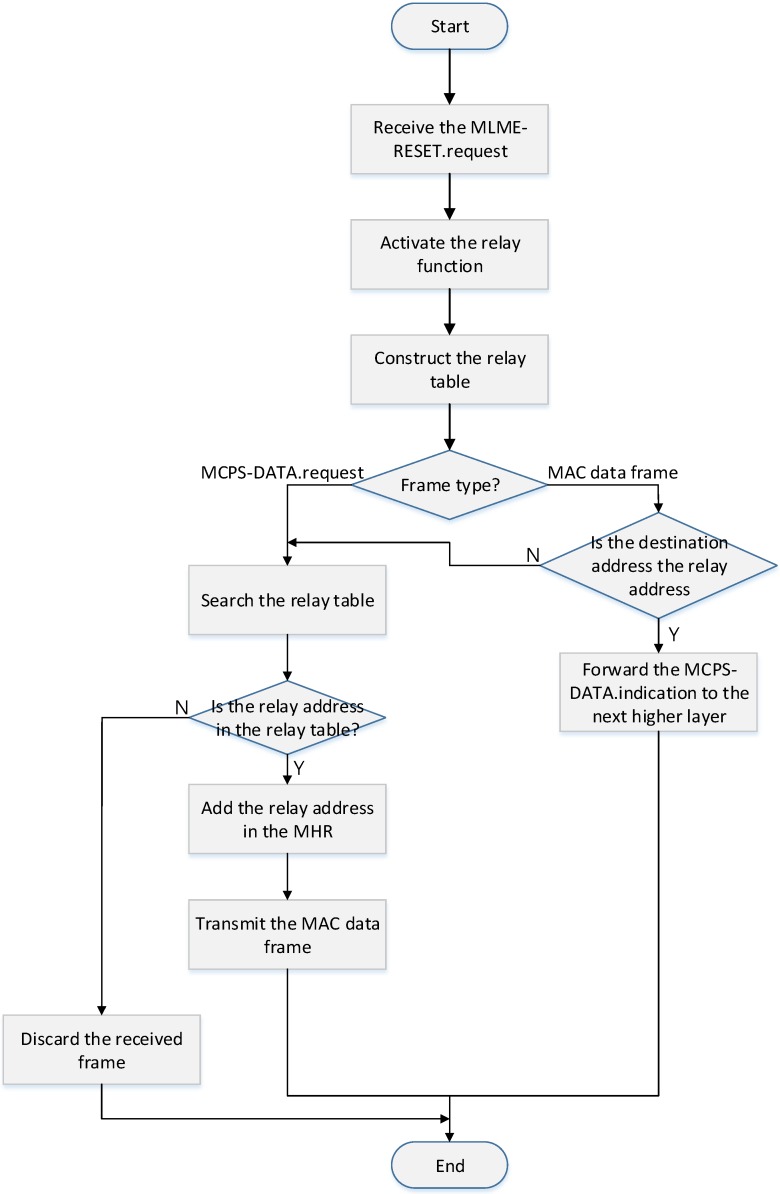
The flow chart for the proposed relay scheme.

After receiving the data frame, the MAC layer of the coordinator ascertains if the relay address field in the data frame indicates its address. If the relay address field in the data frame is equal to its address, it searches its relay table to find the coordinator related to the destination device. Then, it adds an address of the selected coordinator to the addressing field. In Figure 16, because the next device is the PAN coordinator, the coordinator set the relay address field to the PAN coordinator address and transmits the received data frame to the PAN coordinator. Following receipt of the data frame, the PAN coordinator transmits the ACK frame to the MAC layer of the coordinator. The MAC layer of the PAN coordinator ascertains if the relay address and the destination address fields in the data frame indicates its address. If the relay address and the destination address fields are equal to its address, the PAN coordinator transmits the MCPS-DATA.indication message to the higher layer. Otherwise, it discards the received data frame. Figure 19 shows the flow chart for the proposed relay scheme.

As shown in Figure 19, when the coordinator powers on, the MAC layer of the coordinator receives MLME-SET.request message set the RelayFunction parameter to ‘TRUE’ from its higher layer. The MAC layer, after receiving the MLME-SET.request message, activates relay function and constructs a relay table.

When the coordinator receives a frame from its higher layer or other devices, it ascertains a message type. If it receives the MCPS-DATA.request message from its higher layer, the MAC layer searches the relay table. And then, after it adds the relay address to the addressing field, it transmits a data frame to a relay device.

If the coordinator receives a frame from other devices, the MAC layer ascertains if the relay address field and the destination address field in the received data frame indicates its address. If the values of the relay address field and the destination address field are equal to its address, it constructs the MCPS-DATA.indication message and transmits it to its higher layer.

If the value of the relay address field in the received frame is equal to the coordinator address and the value of the destination address field is not equal to the coordination address, the MAC layer selects the relay coordinator and transmits the received data frame to the selected device.

## 4. Performance Evaluation

We conducted the simulation to evaluate the performance of the proposed protocol, comparing with other protocols. We used OMNet++ [30] as the platform for simulation. OMNeT++ is a public source, component based, and open architecture simulation platform with GUI support. It is very suitable for simulating wireless sensor networks owing to its modular structure and using NED language for simple simulation environment configuration. The IEEE 802.15.4 model in [31] is good and was used in several papers to evaluate the performance of their proposed scheme. This model applies to the latest IEEE Std. 802.15.4-2006 standard and implements the GTS mechanism as well as energy model.

To evaluate the performance of the proposed scheme, we modified an INETMANET framework of a model of IEEE 802.15.4 developed for OMNeT++ [31]. Our simulation model consists of the following modules: application layer implementing the traffic generator, Battery module, Network module and Physical layer module. OMNeT++ can set the environment parameter by adjusting the variables in the omnetpp.ini configuration file. The simulations are operated in beacon-enabled mode, and all packets require ACK frame. Many previous works [32,33,34,35] have analyzed the performance of the 802.15.4 MAC protocol in a star network under the assumption that sensor nodes are always active (i.e., power management is disabled). However, in this paper, we analyze the performance of MAC protocol in the cluster tree topology under the assumption that sensor nodes utilize the power management.

In this simulation, we consider a cluster tree topology with a PAN coordinator, a variable number of coordinators and a set of leaf nodes (which are assumed to be the traffic sources). This network configuration could correspond to a realistic scenario of sensor network in which the leaf nodes (the sensors) consist of simple RFD end devices while coordinators could be more complex mains powered FFD nodes. In a real scenario, the PAN coordinator is in charge of programming and communicating the MAC parameters that regulates the beacon emission of the intermediate coordinators, mainly the beacon order of the whole network and the superframe orders of the coordinators. To simplify this procedure in our implementation, the configuration of these MAC parameters is defined through the file omnetpp.ini. Also, following the structure of the original code, a value for the BO and the SO is defined for every node in this file.

In this simulation, we use a constant bit rate traffic generator for synthetic scenarios. All messages are always sent by all nodes to the PAN coordinator (uplink traffic). The data payload size transmitted by each leaf node is fixed at 50 bytes. Each leaf node generates data packets at a rate of one packet per second, and the data rate of each leaf node is fixed at 250 kbps. Also, in this simulation, we considered a frame error rate of approximately 10%. The radio propagation model was two- way ground; the transmission range was set to 15 m, while the carrier sensing range was set to 30 m. In this simulation, it was considered a network where various sensor nodes are placed in a 100 × 100 m area.

In this simulation, we compare the proposed protocol with the cluster-tree protocol of IEEE 802.15.4 [1] and the Adaptive Staggered sLEEp Protocol (ASLEEP) protocol [25]. To evaluate the ASLEEP protocol, we apply the ALEEP protocol to the IEEE 802.15.4 protocol. In the cluster-tree protocol of IEEE 802.15.4, the active periods of parent nodes are scheduled in TDMA, so that only a single parent and its children are active at the same time in the network. In the ASLEEP scheme, each parent node can have a different duration for its active period, depending on the traffic/channel conditions. As a consequence, nodes at the same level in the routing tree can wake up and go to sleep at different times. Clearly, factors such as the node density, the contention, collisions, and the tree level have a significant impact on the network performance. Therefore, we consider these factors in our simulation.

In this simulation, we consider two scenarios to evaluation the proposed scheme. In both scenarios, the PAN coordinator transmits a data frame to a leaf node. The target node is randomly selected among network nodes. In the proposed scheme, the PAN coordinator transmits the data frame to a neighbor coordinator in the RP and then the coordinator forwards a data frame to the leaf node in the GTS without the contention. In the two legacy protocols, the PAN coordinator transmits the data frame to a neighbor coordinator in its active period. Then, the coordinator transmits the data frame to the leaf node in its active period. In the first scenario, to configure the cluster tree, we implemented a simple tree formation algorithm based on the hop count 2, and in the second scenario, we evaluate the network performance according to the increase of the tree depth.

In this simulation, we define that the delivery ratio is the ratio between the number of messages correctly received by the destination and the number of messages sent by the PAN coordinator. And we define that the average energy consumption is the average energy consumed by an intermediate coordinator in the network. We also define that the average latency is the average value of the latency measured from the instant at which the PAN coordinator sends a message to the instant at which the destination node correctly receives the same message.

To evaluate the consumed energy, the energy model of the CC2630, which is a single chip 2.4 GHz IEEE 802.15.4 compliant RF transceiver [36] is used. The energy model in [36] defines four modes for the radio: transmitting, receiving, idle and sleep modes. The energy consumption is calculated by calculating the time spent on radio in each state multiplied by the energy consumption in that mode. Because the CPU consumption is very low compared to energy consumption by the radio [31], we do not consider the CPU consumption in this simulation. The common simulation parameters are summarized in Table 2.

### 4.1. Scenario 1–The Cluster Tree Topology with the Fixed Depth(=2)

**Figure 20:**
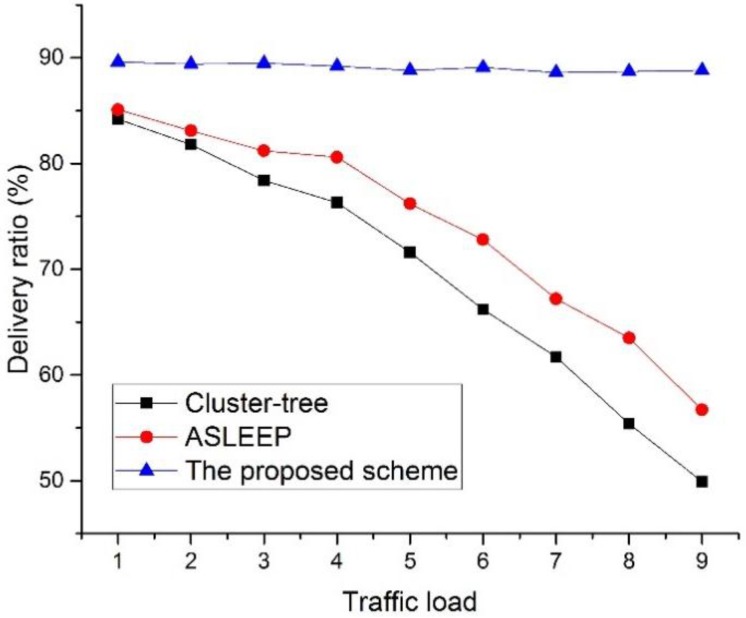
The delivery ratio versus the traffic load in the network.

Figure 20 shows the delivery ratio as a function of the traffic load in the network.

**Figure 21:**
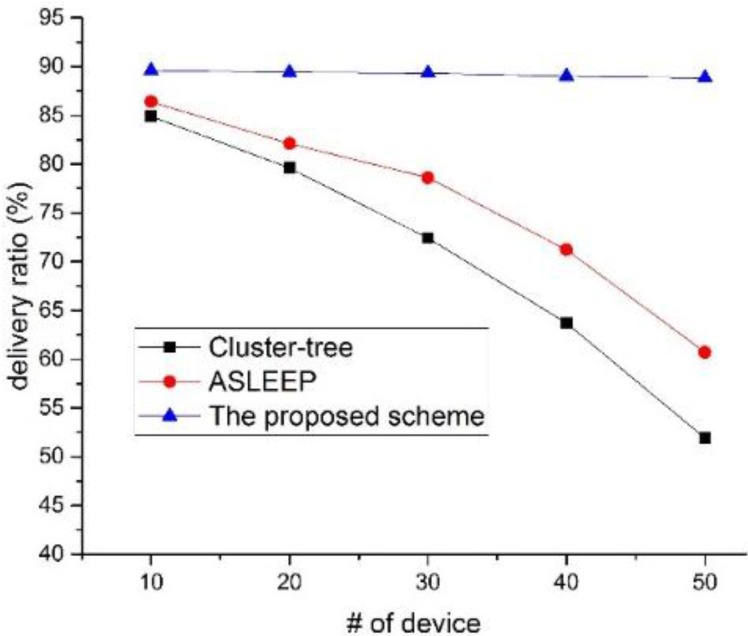
The delivery ratio versus the number of devices in the network.

In this simulation, we define the traffic load to the number of data frames transmitted by devices during the BI. In this simulation, all leaf nodes transmit data frames to the PAN coordinator, and the PAN coordinator transmits data frames to the selected leaf node. Also, we fixed the number of devices in the network to 30. As shown in Figure 20, the delivery ratio of the cluster-tree protocol and the ASLEEP protocol decrease as the traffic load on the network increases. This result is related to the fact that the contention for the channel access and the collision probability increases as the traffic load on the network increases. In Figure 20, because the ASLEEP protocol can slightly adjust the superframe duration within the limited bounds, it can provide the transmission probability higher than the cluster-tree protocol. Therefore, the ASLEEP protocol provides the performance of the delivery ratio higher than the cluster-tree protocol. However, as the traffic load increases, the superframe duration of the ASLEEP protocol reaches the envelope and cannot avoid the increased competition. Thus, the performance of the ASLEEP protocol declines largely. Meanwhile, in the proposed scheme, a coordinator does not contend with other leaf nodes for the channel access since it relays data frames to the next hop coordinator in the CFP. Therefore, the proposed scheme shows the similar delivery ratio regardless of an increase of the traffic load, and we can show that the delivery ratio of the proposed scheme is higher than two legacy protocols. Figure 21 shows the delivery ratio as a function of the number of devices in the network.

In this simulation, the traffic load in the network is fixed to 5. As shown in Figure 21, when the number of devices in the network increases, the delivery ratio of two legacy protocol decreases. This result is because the probability of correct delivery to the next hop reduces due to the increased collision probability. The ASLEEP protocol provides the transmission probability higher than the cluster-tree protocol, but it cannot remove the increased contention. Figure 21 shows that the proposed scheme provides the performance of the delivery ratio higher than two legacy protocols regardless of the node density. In the proposed scheme, when the coordinator relays data frames to the next hop device in the proposed protocol, it transmits data frames in CFP. Thus, it does not contend with other devices for the channel access and is influenced less by the node density. Therefore, the proposed scheme provides better delivery ratio than two legacy protocols.

**Figure 22:**
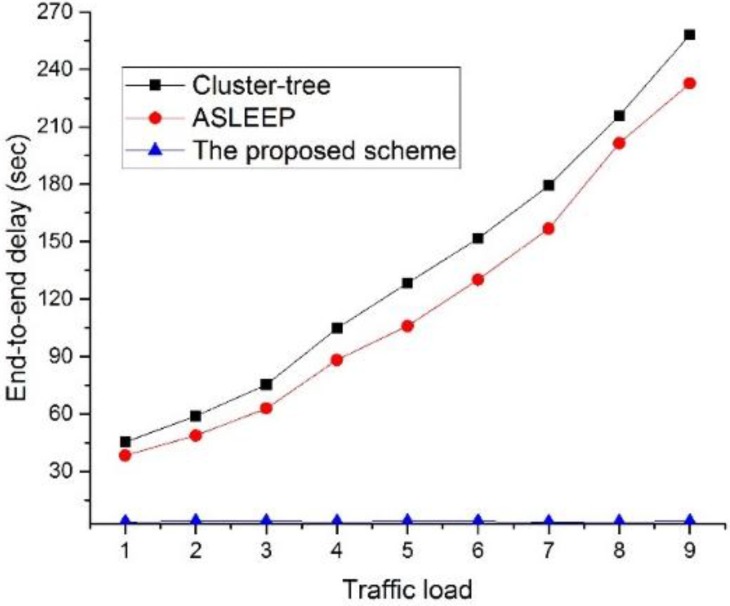
The end-to-end delay as a function of the traffic load in the network.

Figure 22 shows the end-to-end delay as a function of the traffic load in the network.

In this simulation, the number of devices in the network is fixed to 30. As illustrated in Figure 22, when the traffic load on the network increases, the end-to-end delay of two legacy protocols also increases. This result is related to the fact that messages are queued at each parent node for the duration of the active period before they can be forwarded up to the tree. In other words, in two legacy protocols, when the traffic load on the network increases, the contention among devices is intense for the channel access, and the possibility to relay data frame in the same superframe is lower. However, the ASLEEP protocol can slightly regulate the superframe duration, and it can reduce the end-to-end delay somewhat. Meanwhile, in the proposed scheme, because the coordinators can relay data frames to the next hop devices in CFP, it can provide the constant end-to-end delay regardless of the traffic load. Therefore, the end-to-end delay of the proposed scheme is superior to two legacy protocols.

**Figure 23:**
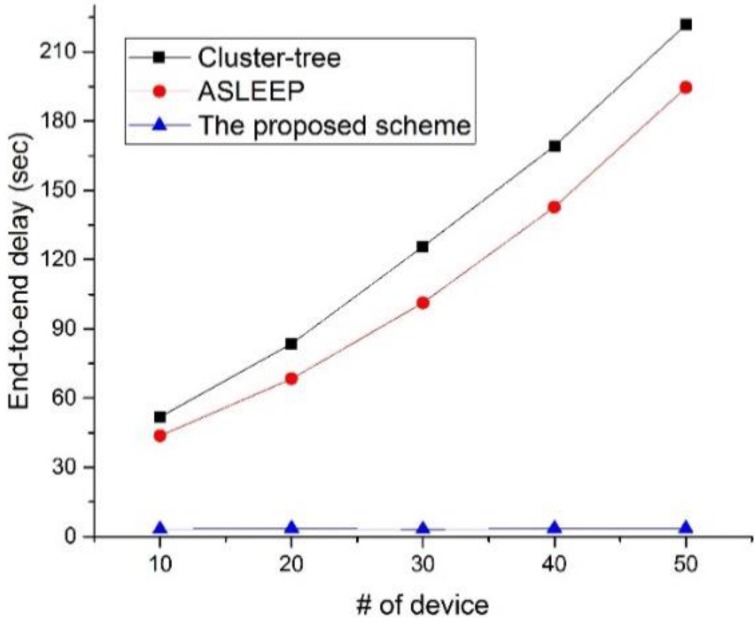
The end-to-end delay as a function of the traffic load in the network.

Figure 23 shows the end-to-end delay as a function of the number of devices in the network.

In this simulation, the traffic load in the network is fixed to 5. In Figure 23, the end-to-end delay of two legacy protocols increases as the node density increases. Because the ASLEEP protocol can adjust the length of the active period and can react the increase of the node density, it shows the superior end-to-end delay performance to the cluster-tree protocol. However, when the node density increases largely, like the cluster-tree protocol, the end-to-end delay of the ASLEEP protocol largely increases due to the contention among devices. However, in the proposed scheme, because the coordinator using the proposed scheme can relay the data frame without the contention, it can provide the constant end-to-end delay regardless of the node density in the network. Therefore, the performance of the proposed scheme is superior to two legacy protocols.

**Figure 24:**
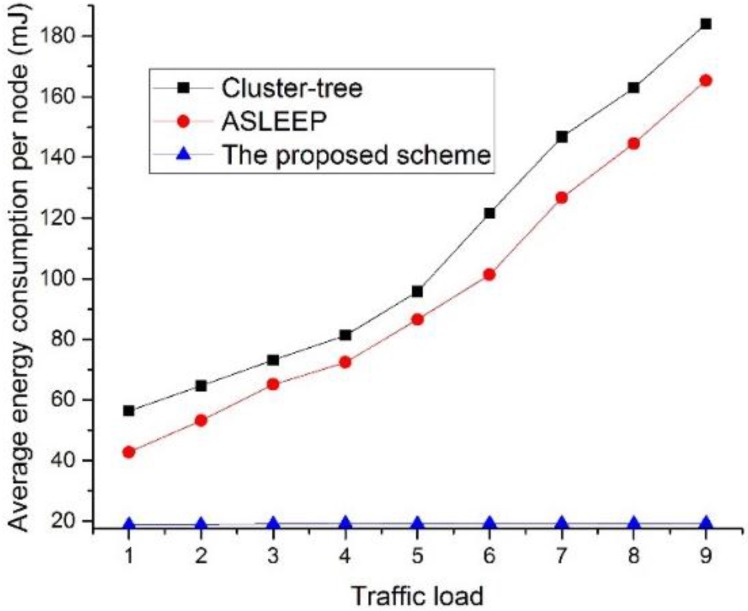
The energy consumption as a function of the traffic load in the network.

Figure 24 shows the energy consumption as a function of the traffic load in the network.

In this simulation, the number of devices in the network is fixed to 30. In Figure 24, when the traffic load increases in two legacy protocols, the energy consumption also increases. This result is because the increase of the traffic load causes the increase of the contention and the collision probability. However, in the proposed scheme, because the coordinator using the proposed scheme can relay data frames to the next hop devices without the contention for the channel access, it can avoid the collision among devices. Therefore, the proposed scheme shows the constant energy consumption performance regardless of the traffic load and provides better performance than two legacy protocols.

**Figure 25:**
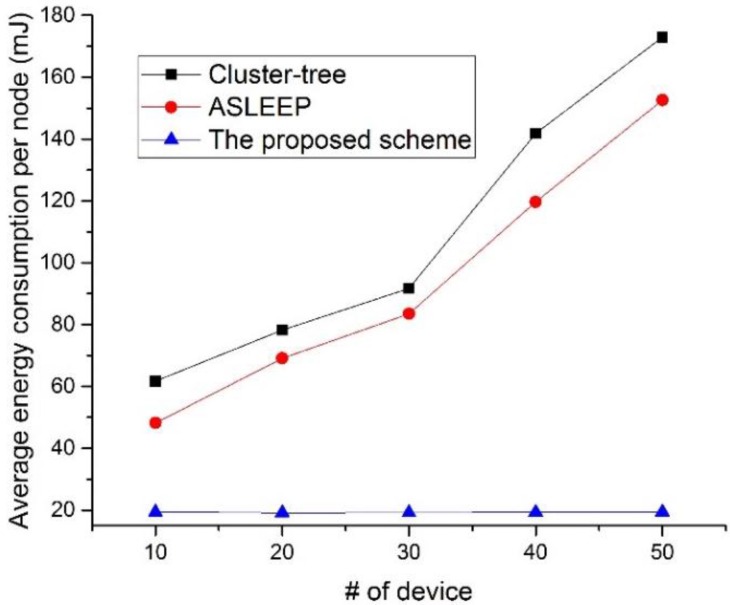
The energy consumption as a function of the number of devices in the network.

Figure 25 shows the energy consumption as a function of the number of devices in the network.

In this simulation, the traffic load in the network is fixed to 5. As shown in Figure 25, the energy consumption of the device using the IEEE 802.15.4 cluster-tree scheme and the ASLEEP scheme increases as the number of devices in the network increases. This result is because the contention overhead for the channel access increases and the channel listening occurred by the contention or the retransmission by the collision increases as the number of nodes in the network increases. Thus, the energy consumption of the device that uses the IEEE 802.15.4 standard or the ASLEEP scheme increases in proportion to the number of nodes. However, in the proposed scheme, the energy consumption of the device that uses the proposed scheme is not influenced by the number of devices in the network since it can transmit the real-time data without the contention for channel access. Therefore, the energy consumption of the proposed scheme is superior to the energy consumptions of two legacy protocols.

### 4.2. Scenario 2–The Cluster Tree Topology with the Variable Depth

**Figure 26:**
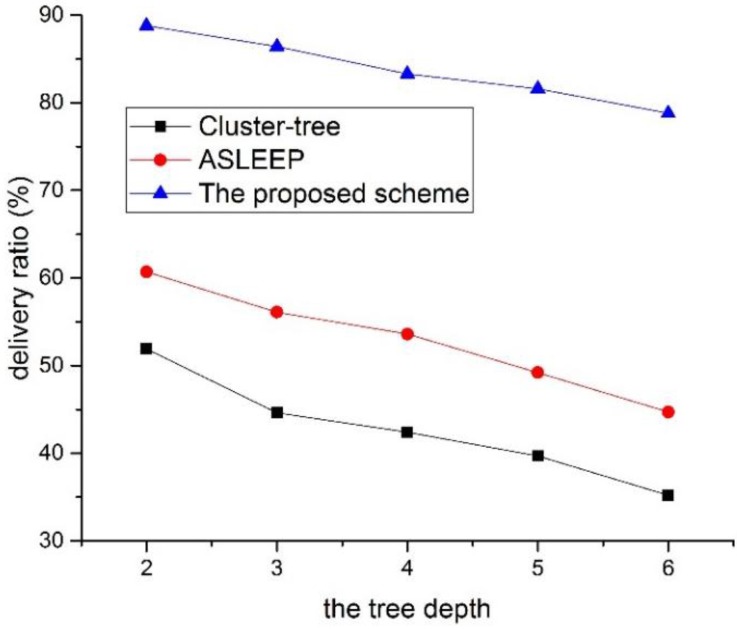
The delivery ratio as a function of the tree level in the network.

The tree depth is one of the major factors that influence the network performance. Therefore, in this subsection, we evaluate the network performance of three protocols according to the tree depth. In this simulation, the traffic load in the network is fixed to 5, and the number of devices in the network is fixed to 50. Figure 26 shows the delivery ratio as a function of the tree level in the network.

As shown in Figure 26, the delivery ratio of three protocol decrease as the tree level of the network increases. In particular, the network performance of two legacy protocols decreases largely. This result is related to the fact that the contention for the channel access and the frame error probability increases as the tree level of the network increases. In Figure 26, because the ASLEEP protocol can slightly adjust the superframe duration within the limited bounds, it can provide the transmission probability higher than the cluster-tree protocol. Therefore, the ASLEEP protocol provides the performance of the delivery ratio higher than the cluster-tree protocol. However, because coordinators in the ASLEEP protocol also contend with other leaf nodes, the frame error rate or the collision increases according to the increase of the tree level. Thus, the performance of the ASLEEP protocol decreases largely. Meanwhile, in the proposed scheme, a coordinator does not contend with other leaf nodes for the channel access since it relays data frames to the next hop coordinator in the CFP. However, the frame error rate of the end-to-end link increases as the tree level of the entire network increases. Therefore, the delivery ratio of the proposed scheme is inversely proportional to the tree level of the network, but the proposed scheme is superior to two legacy protocols.

**Figure 27:**
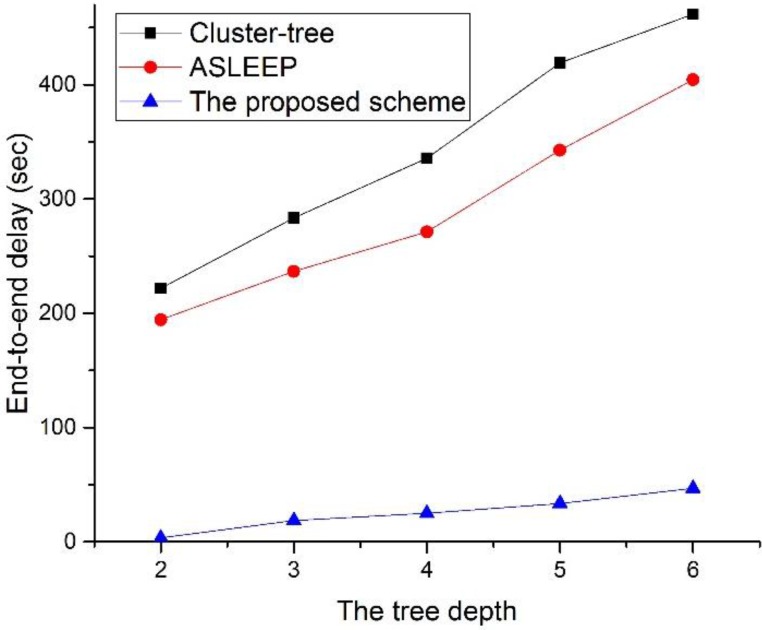
The end-to-end delay as a function of the tree level in the network.

Figure 27 shows the end-to-end delay as a function of the tree level in the network.

As illustrated in Figure 27, when the tree level of the network increases, the end-to-end delay of three protocols also increases but the end-to-end delay of the proposed protocol does not increase greatly. This result is related to the fact that messages are queued at each parent node until they can be forwarded to the next coordinator or the destination node. In other words, in two legacy protocols, when the tree level of the network increases, the contention among devices and the frame error rate increase, and the possibility to relay data frame in the same superframe is lower. Meanwhile, in Figure 27, as the tree level of the entire network increases, the end-to-end delay of the proposed protocol also increases since the frame error rate of the end-to-end link is proportional to the tree level of the network. However, in the proposed, because the coordinator using the proposed scheme can relay the data frame without the contention, the performance of the proposed scheme is superior to two legacy protocols.

**Figure 28:**
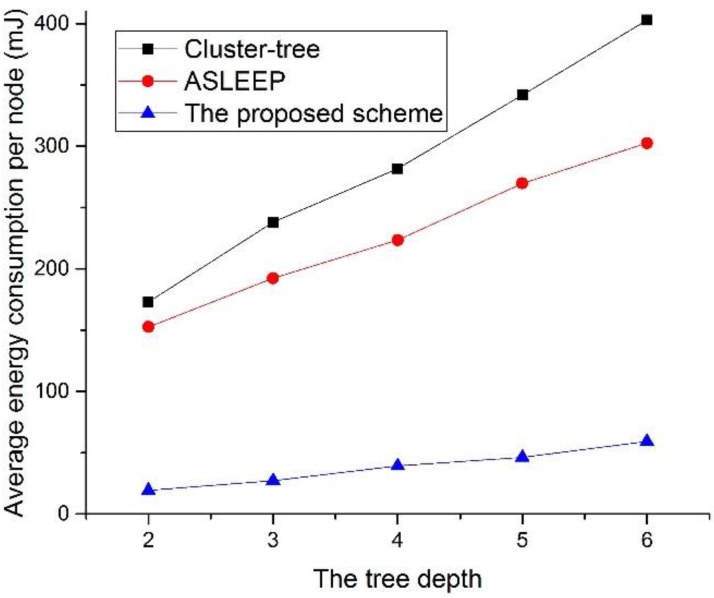
The energy consumption as a function of the tree level in the network.

Figure 28 shows the energy consumption as a function of the tree level in the network.

As shown in Figure 28, apparently, the proposed protocol obtains the lowest energy consumption. In two legacy protocols, the contention overhead for the channel access increases and the channel listening occurred by the contention or the retransmission by the collision increases as the tree level of the network increases. Thus, the energy consumption of the device that uses the IEEE 802.15.4 standard or the ASLEEP scheme increases in proportion to the tree level of the network. Of course, the energy consumption of the proposed scheme is proportional to the increase of the tree level. However, the energy consumption of the proposed scheme is superior to the energy consumptions of two legacy protocols. This is because the retransmission or the transmission attempts by the collision or the frame error of the proposed scheme is the lowest.

## 5. Conclusions

In this paper, we have proposed a new relay transmission scheme to reduce the transmission delay time and the energy consumption in wireless sensor networks. The proposed scheme can improve the flexibility of the network resource allocation and the network coverage in wireless sensor networks. It can also improve the network reliability through the proposed RP allocation. Also, it can configure the enhanced wireless sensor network by reducing the data transmission delay and the power consumption. For this purpose, we proposed the procedure for relay function activation of the MAC sublayer and the procedure for any data frame transmission. The simulation results show that the delay in our protocol is decreased considerably compared to both the IEEE 802.15.4 standard and the ASLEEP scheme, and the delivery ratio is increased largely compared to the IEEE 802.15.4 standard and the ASLEEP scheme. Also, the simulation results show that the energy consumption of the proposed scheme is superior to both the IEEE 802.15.4 standard and the ASLEEP scheme.

## Figures and Tables

**Table 1:** The proposed MLME-SET.request parameter.

Name	Type	Valid Range	Description
PIBAttribute	Octet string	Attribute specific	The name of the PIB attribute to write
PIBAttributeValue	Various	Attribute specific	The value to write to the indicated PIB attribute.
RelayFunction	Boolean	TRUE, FALSE	IF TRUE, the MAC sublayer supports the relay function for data frame transmission. If FALSE, the MAC sublayer doesn‘t support the relay function.

**Table 2:** Simulation Parameters.

Parameters	Value
Radio band	2.4 GHz
Synchronization mode	Beacon-enabled
Carrier sense sensitivity	−85 dBm
PHY Thermal noise	−110 dBm
Channel number	11
Contention Window	2 slots
IEEE 802.15.4 ACK	True
Number of packet retransmissions in case of failure	3
Maximum number of successive backoffs	4
Beacon Order	11
Superframe Order	5
Packet Size	50 bytes
RX current consumption	5.9 mA
TX current consumption	9.1 mA
IDLE current consumption	0.550 mA
Sleep current consumption	0.001 mA

## Abbreviations

The following abbreviations are used in this manuscript:

IoT: Internet of things
WPAN: Wireless personal area network
PHY: Physical
MAC: Media access control
O-QPSK: Offset quadrature phase-shift keying
SD: Superframe duration
BI: Beacon interval
CAP: Contention access period
CFP: Contention free period
RP: Relay period
MHR: MAC header
ED: Energy detection
MLME: MAC layer management entity
MCPS: MAC common part sublayer
ACK: Acknowledgement
ASLEEP: Adaptive staggered sleep protocol
